# The arrangement of *Brachypodium distachyon* chromosomes in interphase nuclei

**DOI:** 10.1093/jxb/erw325

**Published:** 2016-09-01

**Authors:** Ewa Robaszkiewicz, Dominika Idziak-Helmcke, Magdalena A. Tkacz, Kornel Chrominski, Robert Hasterok

**Affiliations:** ^1^Department of Plant Anatomy and Cytology, Faculty of Biology and Environmental Protection, University of Silesia in Katowice, Katowice, Poland; ^2^Institute of Computer Science, Faculty of Material and Computer Science, University of Silesia in Katowice, Sosnowiec, Poland; ^3^Institute of Technology and Mechatronics, Faculty of Material and Computer Science, University of Silesia in Katowice, Sosnowiec, Poland

**Keywords:** *Brachypodium distachyon*, chromosome territories, computer modelling, interphase nucleus, model grass, nuclear architecture.

## Abstract

The spatial organization of chromatin within the interphase nucleus and the interactions between chromosome territories (CTs) are essential for various biological processes, such as DNA replication, transcription, and repair. However, detailed data about the CT arrangement in monocotyledonous plants are scarce. In this study, chromosome painting was used to analyse the distribution and associations of individual chromosomes in the 3-D preserved nuclei of *Brachypodium distachyon* root cells in order to determine the factors that may have an impact on the homologous CT arrangement. It was shown that the frequency of CT association is linked to the steric constraints imposed by the limited space within the nucleus and may depend on chromosome size and morphology as well as on the nuclear shape. Furthermore, in order to assess whether the distribution of interphase chromosomes is random or is subject to certain patterns, a comparison between the experimental data and the results of a computer simulation (ChroTeMo), which was based on a fully probabilistic distribution of the CTs, was performed. This comparison revealed that homologous chromosome arm CTs associate more often than if they were randomly arranged inside the interphase nucleus.

## Highlight

A comparison between experimentally observed and computationally simulated 3-D nuclei of the model grass *Brachypodium distachyon* reveals that the homologous chromosome territories associate more often than if they were randomly arranged.

## Introduction

The recent rapid progress in DNA sequencing technologies has enabled the almost complete nuclear genome sequence to be read for an increasing number of species. However, it has become clear that knowledge about the linear nucleotide sequence itself is not sufficient to understand fully how genomes are organized and modulated *in vivo* within the cell nucleus. Some aspects that have a functional relevance to gene regulation, such as epigenetic modifications, are now the subjects of intensive studies (for recent reviews, see Rothbart and Strahl, 2014; Swygert and Peterson, 2014; Sharma *et al.*, 2015; Venkatesh and Workman, 2015). However, detailed data about the impact of the internal nuclear architecture and the higher order chromatin organization on functional processes inside the nucleus are still missing.

It is common that individual decondensed chromosomes at interphase in all of the eukaryotes studied to date are not spread throughout the nucleus but occupy distinct and specific areas which are called chromosome territories (CTs; Cremer *et al.*, 1982; Manuelidis, 1985; Lichter *et al.*, 1988; Pinkel *et al.*, 1988). CTs are usually compactly organized and their intermingling is absent or limited to the periphery of the territory, which may allow some interchromosomal interactions (Branco and Pombo, 2006). The size and structure of an individual CT primarily depend on factors such as the size of the given chromosome and the transcriptional status of the genes it carries (Croft *et al.*, 1999; Mahy *et al.*, 2002). One of the most intriguing questions about CTs concerns their spatial distribution inside the interphase nucleus—is it random or is it subject to certain patterns? The first indication of a non-random positioning of chromosomes in the nucleus was the observation of the localization of chromosomes 18 and 19 in human lymphocyte cells (Croft *et al.*, 1999; Cremer *et al.*, 2001). Despite the similar size of the analysed chromosome pairs, the gene-rich chromosome 19 was found in the nuclear interior, while the gene-poor chromosome 18 was preferentially located close to the nuclear envelope. This gene density-related radial arrangement in the nucleus was further confirmed by analyses that comprised all human chromosome domains (Boyle *et al.*, 2001). The following studies of different cell types or species showed that CTs may be positioned radially according to either the gene density of a given chromosome (Croft *et al.*, 1999; Bridger *et al.*, 2000; Boyle *et al.*, 2001), their size (Sun *et al.*, 2000; Bolzer *et al.*, 2005), or both of these factors (Tanabe *et al.*, 2002; Mayer *et al.*, 2005; Heride *et al.*, 2010). It is also conserved among different groups of animals, such as primates (Tanabe *et al.*, 2002; Neusser *et al.*, 2007), mice (Mayer *et al.*, 2005), cattle (Koehler *et al.*, 2009), birds (Habermann *et al.*, 2001), amphibians, and reptiles (Federico *et al.*, 2006). Such a conservation during evolutionary history suggests a strong functional significance of this type of nuclear architecture.

A less understood, but equally important, phenomenon regarding genomic organization is the spatial positioning of chromosome territories relative to one another. A side-by-side arrangement of homologues was reported in adult human Sertoli cells (Chandley *et al.*, 1996), non-cycling human fibroblasts (Nagele *et al.*, 1999), and the heterologues in lung-derived fibroblast cell lines (Zeitz *et al.*, 2009). Additionally, a correlation between the association of chromosomes and tissue type was noticed in nuclei of mice (Parada *et al.*, 2004). The spatial arrangements of homologous chromosomes is of particular interest, because some, at least transient, interactions between them are necessary for homologous recombination and DNA repair.

Corresponding studies in plants are extremely limited due to their usually much larger nuclear genome sizes and the less favourable organization of plant chromatin, which is saturated with ubiquitous repetitive DNA (Bennett and Leitch, 2005). However, the side-by-side arrangement of chromosomes was successfully analysed in the dicotyledonous model plant *Arabidopsis thaliana* and in *A. lyrata* (Pecinka *et al.*, 2004; Berr *et al.*, 2006). It has been shown that both homologous and heterologous chromosomes in these species were randomly located inside the nucleus. The only exceptions were the nucleolus-organizing region (NOR)-bearing chromosomes, which demonstrated a more frequent association. The possible explanation of such a phenomenon is that these chromosomes participate in the formation of a single nucleolus.

The direct visualization of CTs became possible in the 1980s along with the development of the chromosome painting methods that are based on fluorescence *in situ* hybridization (FISH) with probes that are specific for entire chromosomes or chromosome arms (Lichter *et al.*, 1988; Pinkel *et al.*, 1988). However, the territorial organization of chromosomes during interphase was initially postulated almost a hundred years earlier by Carl Rabl (1885), who observed a polarized pattern of centromere and telomere distribution during cell division and in the following interphase. Such an organization of chromatin, which was later called the Rabl configuration, was found to be frequent in plants, but was also observed in a few animal species, such as the fruit fly (Hochstrasser *et al.*, 1986), and in both budding and fission yeast (Funabiki *et al.*, 1993; Jin *et al.*, 1998). In plant species, it was suggested that one of the factors that might affect the clustering of centromeres and telomeres at the opposite poles of the nucleus is the size of the nuclear genome. Dong and Jiang (1998) showed that the Rabl configuration is present in some cereal species that have nuclear genomes >4800Mb (wheat, rye, barley, and oats), while it is absent in sorghum and rice, which have rather small genomes (<1000Mb). The organization of centromeres and telomeres in different plant species, such as *Pisum sativum*, *Vicia faba* (Rawlins *et al.*, 1991), *Allium cepa* (Fussell, 1992), as well as in representatives of the *Arabidopsis*, *Brassica* and *Solanum* genera (Harrison and Heslop-Harrison, 1995; Kamm *et al.*, 1995), appear to be in concordance with this hypothesis. However, we recently found the Rabl configuration also in root nuclei of *Brachypodium distachyon* (Idziak *et al.*, 2015), a model grass species with a genome size of ~309Mb/1C DNA (Catalan *et al.*, 2012). Thus, it seems probable that some additional factors, such as chromosome length and/or the mitotic activity of the cells, may influence the presence or absence of centromere and telomere polarization. For instance, analysis of wheat–rye addition lines showed some differences in chromosome organization between meristematic and differentiated nuclei. A less obvious Rabl configuration and round-shaped CTs in differentiated leaf nuclei were observed in the case of both A and B chromosomes of rye (Schubert *et al.*, 2011).

The study on spatial centromere and telomere distribution was also one of the first insights into the *B. distachyon* nuclear architecture. Because the chromosome painting methodology is limited strictly to only a handful of plants with small genomes, including the *Brachypodium* genus (Idziak *et al.*, 2011; Betekhtin *et al.*, 2014), it is of particular interest to complement the data by investigating the behaviour and association of chromosomes during interphase. In this study, we present the results of 3-D FISH with pools of chromosome arm-specific bacterial artificial chromosome (BAC) clones in the root nuclei of *B. distachyon* with the aim of analysing the arrangement of homologous chromosomes during interphase. Additionally, in order to assess whether their distribution is random or is subject to some defined patterns, the experimental data were compared with the results of a computer simulation (ChroTeMo) that assumes a fully probabilistic distribution of interphase chromosomes (Tkacz *et al*., 2016).

## Materials and methods

### Plant material

*Brachypodium distachyon* (L.) P. Beauv seeds of the reference Bd21 genotype, a diploid species with 2*n*=10 chromosomes, were used. The material was obtained from the collection held by USDA-NPGS.

### Chromosome preparations

Chromosome preparations of the root meristems were done according to a previously described procedure (Jenkins and Hasterok, 2007). The seeds were germinated in Petri dishes with moistened filter paper at room temperature in the dark for 2–3 d. Seedlings with roots ~1.5–2cm long were collected and immersed in ice-cold water for 24h, fixed in a mixture of methanol/glacial acetic acid in a 3:1 (v/v) proportion, and stored at −20 °C until use.

The fixative was removed by washing the excised roots in a 0.01M citric acid–sodium citrate buffer (pH 4.8) for 15min. Then, the roots were digested in an enzyme mixture comprising 20% (v/v) pectinase (Sigma-Aldrich), 1% (w/v) cellulase (Sigma-Aldrich), and 1% (w/v) cellulase ‘Onozuka R-10’ (Serva) for 1h 15min at 37 ºC. After digestion, the meristems were dissected from the root tips, squashed in 45% acetic acid, and frozen on dry ice.

### Preparation of isolated nuclei

The isolation of the nuclei was carried out according to Dolezel *et al.* (1989). The seeds were grown in Petri dishes as described above. Each step of the following procedure was performed on ice. After obtaining roots of the appropriate length, whole seedlings were fixed in 4% formaldehyde in 1× phosphate-buffered saline (PBS) (pH 7.3) for 30min and then washed three times in 1× PBS (10min each). Then, the roots were cut from the seedlings and washed for 20min in a TRIS buffer (10mM Tris–HCl, pH 7.5, 10mM Na_2_-EDTA, 100mM NaCl). Next, the roots were chopped in an LB01 buffer (15mM Tris–HCl, pH 7.5, 2mM Na_2_-EDTA, 0.5mM spermine ·4HCl, 80mM KCl, 20mM NaCl, 0.1% Triton X-100, 15mM β-mercaptoethanol) with a razor blade in a Petri dish. The suspension with the isolated nuclei was filtered through a mesh filter with a pore size of 30 µm and dropped onto microscopic slides cooled to 0 °C. After air-drying, the slides were stored at −20 °C until use.

### Immunodetection of fibrillarin in the nuclei

Fibrillarin was immunostained according to the method described by Jasencakova *et al.* (2001) to visualize the nucleoli in the isolated nuclei. Briefly, the anti-fibrillarin mouse monoclonal IgG1 primary antibody (1:100 dilution in 1% BSA in PBS; Novus Biologicals Cat. no. NB300-269) was used. As secondary antibody, Alexa Fluor 488 goat anti-mouse IgG (1:200 dilution in 1% BSA in PBS; Invitrogen, Molecular Probes, Cat. no. A-11001) was applied.

### DNA probes and fluorescence *in situ* hybridization

The BAC clones used as the painting probes were obtained from two genomic DNA libraries of *B. distachyon*, BD_ABa and BD_CBa, which were constructed and described earlier (Febrer *et al.*, 2010; International Brachypodium Initiative, 2010). To eliminate unspecific hybridization signals, clones containing >30% of repetitive sequences were excluded from the painting pools. The selection of the BAC clones was performed using bioinformatic methods (Febrer *et al.*, 2010) and their specificity as single-locus probes was confirmed by Idziak *et al.* (2011). To obtain clear, specific signals of hybridization with the pools of BACs delivered from chromosome 3 (Bd3), the content of the repeats of the selected clones was further restricted to 22%. The list of the BAC clones that were used to paint individual chromosomes is provided in Supplementary Tables S1–S5 at *JXB* online.

The selected BACs were divided into pools of 6–10 clones each, as described in Idziak *et al.* (2011), and isolated together using a standard alkaline method. The BAC DNA was labelled by nick translation with digoxigenin-11-dUTP (Roche) for short chromosome arms, and with tetramethylrhodamine-5-dUTP (Roche) for long arms. The details of probe labelling and the following FISH procedure were as described previously (Idziak *et al.*, 2011) with some modifications. After treatment with RNase, the preparations of isolated nuclei were treated with pepsin (100 µg ml^–1^ in 0.01M HCl; Sigma) for 10min at 39 °C to remove the excess cytoplasm, post-fixed in 1% formaldehyde in 1× PBS, dehydrated in an ethanol series, and air-dried. The BAC DNAs were precipitated and resuspended in a hybridization mix containing 50% deionized formamide and 10% dextran sulphate in 2× SSC (saline sodium citrate). The hybridization mix was pre-denatured for 10min at 75 °C, applied to the slides, and denatured together at 75 °C for 4.5min before incubation in a moist chamber at 37 °C for ~40h. After hybridization, the slides were washed in 10% formamide in 2× SSC (2×4min, 42 °C), which is equivalent to a 79% stringency. The hybridization signals of the digoxigenin-labelled BACs were detected using fluorescein isothiocyanate (FITC)-conjugated anti-digoxigenin antibodies (Roche). The chromosomes were counterstained with 2.5 µg ml^–1^ DAPI in Vectashield.

### Image acquisition and processing

All of the images of the interphase nuclei with probes that were specific to Bd2, Bd3, Bd4, and Bd5, as well as those after the immunodetection of fibrillarin, were acquired using an Olympus FV1 000 confocal microscope system equipped with a 60×/1.35 PlanApo objective. The images of the interphase nuclei with Bd1 and Bd2 and mitotic preparations were acquired using a Zeiss Axio Imager.Z.2 wide-field fluorescence microscope equipped with an AxioCam Mrm monochromatic camera and the Apotome.2 system. Image stacks were acquired by traversing from the top to the bottom of a nucleus in 200–250nm steps. Image processing, including the rendering of the *Z*-stacks from a series of optical sections of the nuclei, was performed using ImageJ (Wayne Rasband, National Institutes of Health, USA) or Imaris (Bitplane) software. Imaris was also used to construct the 3-D models of the analysed nuclei using the ‘Contour surface’ wizard. These models were also utilized to measure the volume of the nuclei and nucleoli.

### Computer simulation of random CT distribution

In order to assess the 3-D topology of the interphase chromosomes inside the *B. distachyon* nuclei, experimental data were compared with the predictions that were computed by the Chromosome Territory Modeller (ChroTeMo)—a script for modelling CTs that assumes a random distribution of the interphase chromosomes inside the nucleus, which was designed by us for this study (Tkacz *et al*., 2016). The model used in our study simulates the process of chromosome decondensation at the end of mitosis/the beginning of the G_1_ phase. As decondensed chromosomes appear to be built up from the ~1Mb domains that are 400–800nm in diameter (Visser *et al.*, 2000; Albiez *et al.*, 2006), in our simulation they were rendered as a chain of beads whose size corresponded to the size and DNA content of the domains (1Mb of DNA, 500nm diameter). In the initial step of the simulation process, after the creation of the nucleus and nucleolus (according to previously set parameters), the position of the centromeres inside the nucleus (but outside the nucleolus) was randomly chosen and centromeres were drawn as beads. Then, the chromosomes in their condensed state were built as chains of beads, and the number of beads in a single chain corresponded to the length of a given *B. distachyon* mitotic chromosome. The *B. distachyon* karyotype consists of five pairs of chromosomes that range in length from 3.5 µm to 7 µm at the somatic metaphase (Garvin *et al.*, 2008), so the chains of beads representing them included 7–14 beads. In the final step, the subsequent beads were added to the already drawn beads, randomly chosen, in order to reflect the decondensation process until the entire space was uniformly filled with simulated chromatin fibres. The number of beads comprising a single chromosome corresponds to its length in Mb (75, 59, 60, 48, and 28 for Bd1–Bd5, respectively; International Brachypodium Initiative, 2010). Our model also takes into account the morphology of the chromosomes, in particular their arm length and the position of the centromeres.

The other software developed by us, a Chromosome Territory Viewer (ChroTeVi), was used to colour and visualize individual homologous chromosomes using the data that were generated by ChroTeMo. ChroTeVi enables the precise analysis of CT distribution due to the possibility of viewing only the selected chromosome pairs, while the rest of the chromatin is transparent. Moreover, it also has the ability to rotate the generated image and to zoom in and out (Tkacz *et al.*, 2016).

The model was run 115 times and the minimal distances between the modelled homologous CTs were measured. In both the experimental and simulated data, two domains were considered to be associated when their edges were closer than 500nm to each other. A comparison of the experimentally obtained values with the simulated values was performed, and a statistical analysis using Pearson’s χ^2^ test of goodness of fit was performed. The differences were considered to be significant at the *P<*0.001 level.

## Results

### *B. distachyon* root nuclei vary significantly in shape and size

The computer simulation of the random distribution of chromosome territories in the nucleus that was used in this study requires initial parameters, which were experimentally measured. One of these was the size of the sphere representing the nucleus. To determine this parameter, 100 nuclei isolated from *B. distachyon* roots and counterstained with DAPI were analysed (Table 1). This revealed that the nuclear shapes were remarkably diversified and included spherical, elongated, and very long, rod-shaped nuclei. The population of the nuclei was divided into three categories based on the length (l) to width (w) ratio. The l and w parameters of the nuclei in the *x*:*y* plane were measured and each nucleus was classified as spherical (l/w=1.0–1.4), elongated (l/w=1.5–3.5), or rod-shaped (l/w >3.5). Spherical nuclei were observed with the highest frequency (42%), elongated nuclei constituted 34% of the population, while the rod-shaped nuclei were less common (24%). Additionally, a considerable diversity of nuclear volumes, which were estimated using Imaris software, was also observed, regardless of the category of the nucleus. The elongated nuclei were the most diversified and had a nuclear volume that ranged from 76.2 µm^3^ to 1122 µm^3^, while the rod-shaped nuclei were more similar to each other (68.5–397 µm^3^). However, despite the significant heterogeneity, the mean volumes of the nuclei in each category were similar with ~150 µm^3^ (Table 1).

**Table 1.** Dimensions and volumes of different nuclei types in *B. distachyon* roots (100 nuclei analysed)

Nuclear shape	Frequency of nuclear shape category (%)	Mean axis length (µm)			Minimal–maximal volume (µm^3^)	Mean volume (µm^3^)	Median volume (µm^3^)
		*x*	*y*	*z*			
Spherical	42	6.9	5.7	8.1	55.5–475.0	165.1	161.0
Elongated	34	10.6	5.1	8.3	76.2–1122.0	145.8	187.0
Rod-shaped	24	13.7	2.7	6.6	68.5–397.0	157.0	142.0

The other parameter that is required for a CT simulation using ChroTeMo is the volume of the nucleolus calculated as a percentage of the nuclear volume. In order to visualize the nucleoli inside the DAPI-counterstained nuclei, the immunodetection of fibrillarin, which is one of the nucleolus-specific proteins, was performed. The majority of the nuclei (88%) contained only one nucleolus, which was visible as a green sphere and its localization coincided with the DAPI-negative area inside the nucleus. The volumes of both the nuclei and nucleoli were again estimated using the structures modelled in Imaris that were based on the experimentally obtained images. The absolute volume of the nucleoli varied from 2 µm^3^ to 79 µm^3^, while the volumes of the nucleoli expressed as a percentage of the nuclear volume ranged from 2% to 34%, with most of the nucleoli (68%) being below 10% (Supplementary Fig. S1). The examples of two nuclei with similar volumes but with different nucleoli sizes are presented in Supplementary Fig. S2 and Supplementary Videos S1A, B.

### The arrangement of homologous CTs depends on chromosome length and morphology

**Fig. 1.**
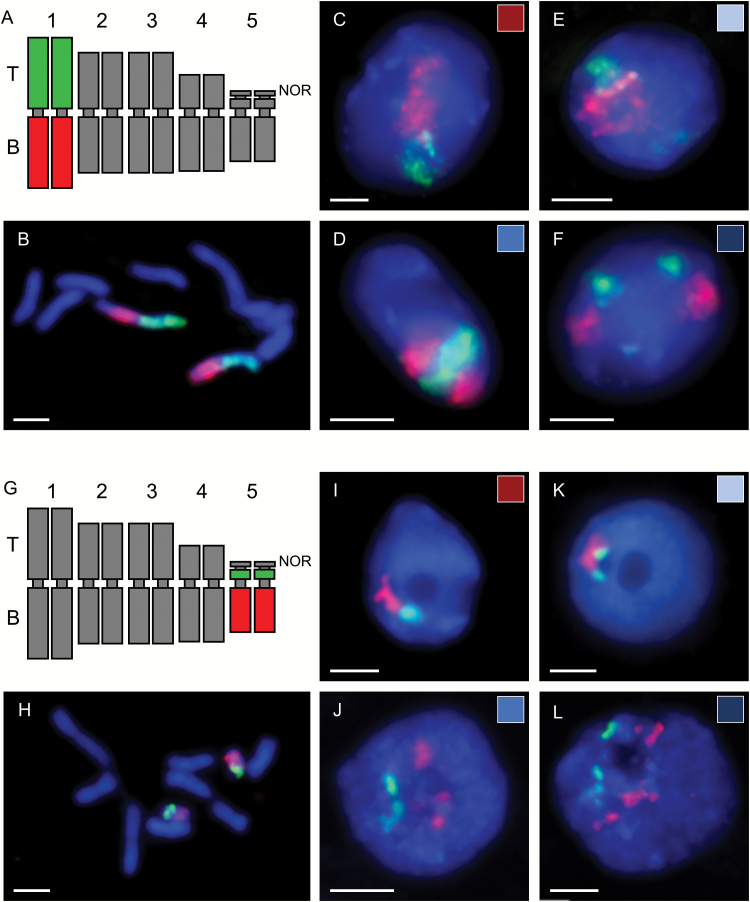
Arrangement of Bd1 (A–F) and Bd5 (G–L) homologous CTs in the nuclei of *B. distachyon* (200 nuclei analysed for Bd1 and 96 nuclei for Bd5). (A, G) Idiograms showing the differential labelling of the top (green) and bottom (red) arms of chromosomes. (B, H) Somatic chromosome preparations used as a control to verify the specificity of the probes. (C, I) Complete association of homologues. (D, J) Association of only top arm CTs. (E, K) Association of only bottom arm CTs. (F, L) Complete separation of homologues.The colour of the rectangle in the upper right side of each photomicrograph corresponds to the sector of the pie chart representing the particular association type in Fig. 2. DAPI counterstaining (blue fluorescence); scale bars=2 µm.

To investigate the spatial distribution of the homologous chromosomes in *B. distachyon* root nuclei, a series of FISH experiments was conducted. In each experiment, the probes that painted a single chromosome pair were used. The discrimination between the chromosome arms was achieved by differential labelling with arm-specific BAC contigs (Fig. 1A, G) on isolated nuclei as well as on mitotic metaphase chromosomes. The somatic chromosome preparations were used as a control to verify the specificity of the probes (Fig. 1B, H).

**Fig. 2.**
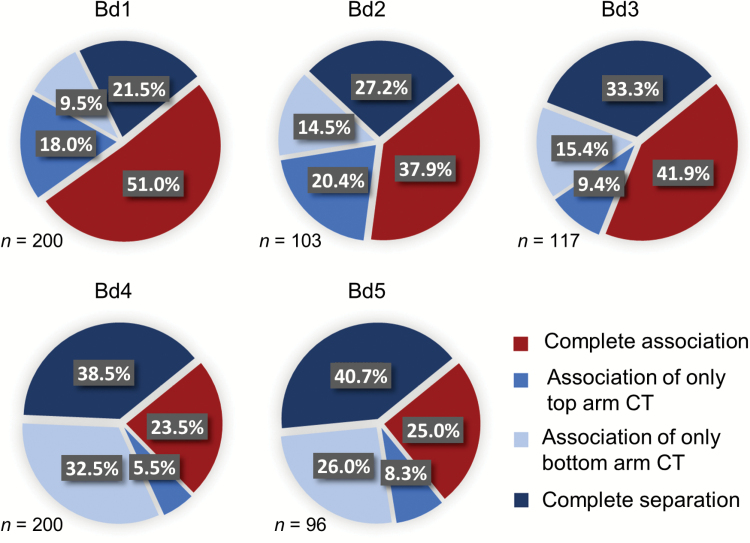
Association frequencies of homologous chromosome arm territories in root nuclei (T, top arm; B, bottom arm; +, CTs associated; –, CTs separated; *n*, number of nuclei analysed for each chromosome pair).

The hybridization of the chromosome-specific probes to *B. distachyon* nuclei revealed the presence of discrete 3-D CTs. The area occupied by each chromosome domain roughly corresponded to the size of the given chromosome; for example, it was noticeably larger in the case of Bd1 as compared with Bd5 (Fig. 1). The territories of homologous chromosomes were arranged in four different configurations from a complete association (Fig. 1C, I) through the association of a single-arm only, top (Fig. 1D, J) or bottom (Fig. 1E, K), to the separation of entire CTs (Fig. 1F, L). The analysis of 100–200 nuclei for each painted chromosome showed that all four types of CT arrangements were observed in the case of each of the chromosomes, but that the frequency of a particular pattern of CT distribution varied depending on the chromosome pair (Fig. 2).

The *B. distachyon* karyotype consists of five pairs of chromosomes, and the metacentric Bd1 constitutes the longest chromosome in the complement. In most of the root nuclei, a pair of Bd1 homologous chromosomes was found to be associated along both arms (51%), while the opposite situation, the complete separation of CTs, was found in 21.5% of the analysed nuclei. Similarly, a close spatial neighbourhood of entire homologues was often observed in the case of two other metacentric chromosomes, Bd2 and Bd3, although the percentage of nuclei that displayed this type of arrangement was lower when compared with Bd1 (37.9% for Bd2 and 41.9%, for Bd3). Correspondingly, the complete separation of the Bd2 and Bd3 homologues was more frequent (27.2% and 33.3%, respectively). In the case of all of the *B. distachyon* metacentric chromosomes, the association of only the top or bottom arms was less frequent than the association or separation of entire homologues. Nonetheless, the close spatial vicinity of the top arm territories was found more often for Bd1 and Bd2, while it was rather rare in the case of Bd3 (9.4% of the nuclei).

The CTs of the two remaining chromosomes Bd4 and Bd5, which are acrocentric, were usually separated in the *B. distachyon* root nuclei. Additionally, they were often found to be associated only by their bottom arms (32.5% and 26.0%, respectively), while the opposite orientation, the close vicinity of the homologue top arms, was very infrequent (5.5% for Bd4 and 8.3% for Bd5). Such a phenomenon is probably linked to the big difference between the length of the top and bottom arms of these particular chromosomes.

The results of the CT distribution analysis in the root nuclei of *B. distachyon* strongly suggest that the length of a particular chromosome may influence the dominant pattern of its spatial arrangement inside the nucleus. It was shown that regarding the three longest chromosome pairs, Bd1–Bd3, the homologous chromosome arm territories were usually associated. In contrast, the homologous CTs of the significantly shorter chromosomes Bd4 and Bd5 were more frequently separated from each other. This hypothesis was confirmed by the Pearson’s χ^2^ test of independence and was found to be highly significant (χ^2^=85.2; *P*<0.001).

### The arrangement of homologous CTs depends on nuclear shape

**Fig. 3.**
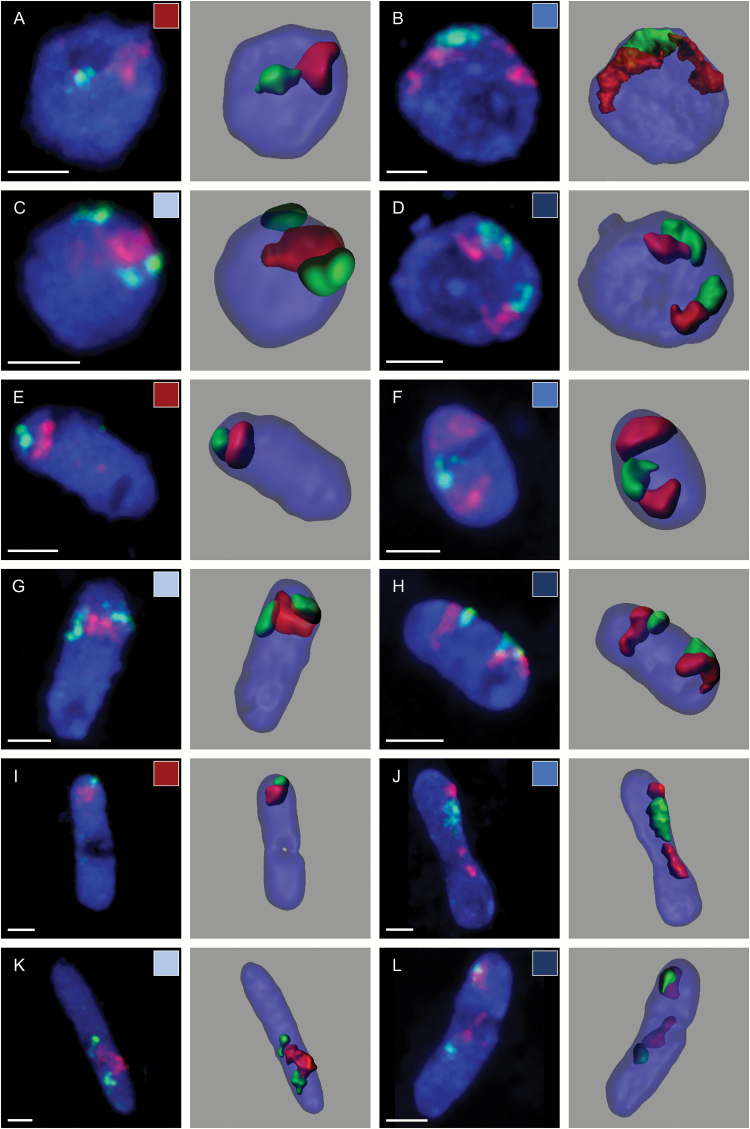
Arrangement of Bd4 homologous CTs in the spherical (A–D), elongated (E–H), and rod-shaped (I–L) nuclei (200 nuclei analysed). (A, E, I) Complete association of homologues. (B, F, J) Association of only top arm CTs. (C, G, K) Association of only bottom arm CTs. (D, H, L) Complete separation of homologues. The nuclei structures modelled with the Imaris software after FISH are presented on the right-hand side of each photomicrograph. DAPI counterstaining (blue fluorescence); scale bars=2 µm.

**Fig. 4.**
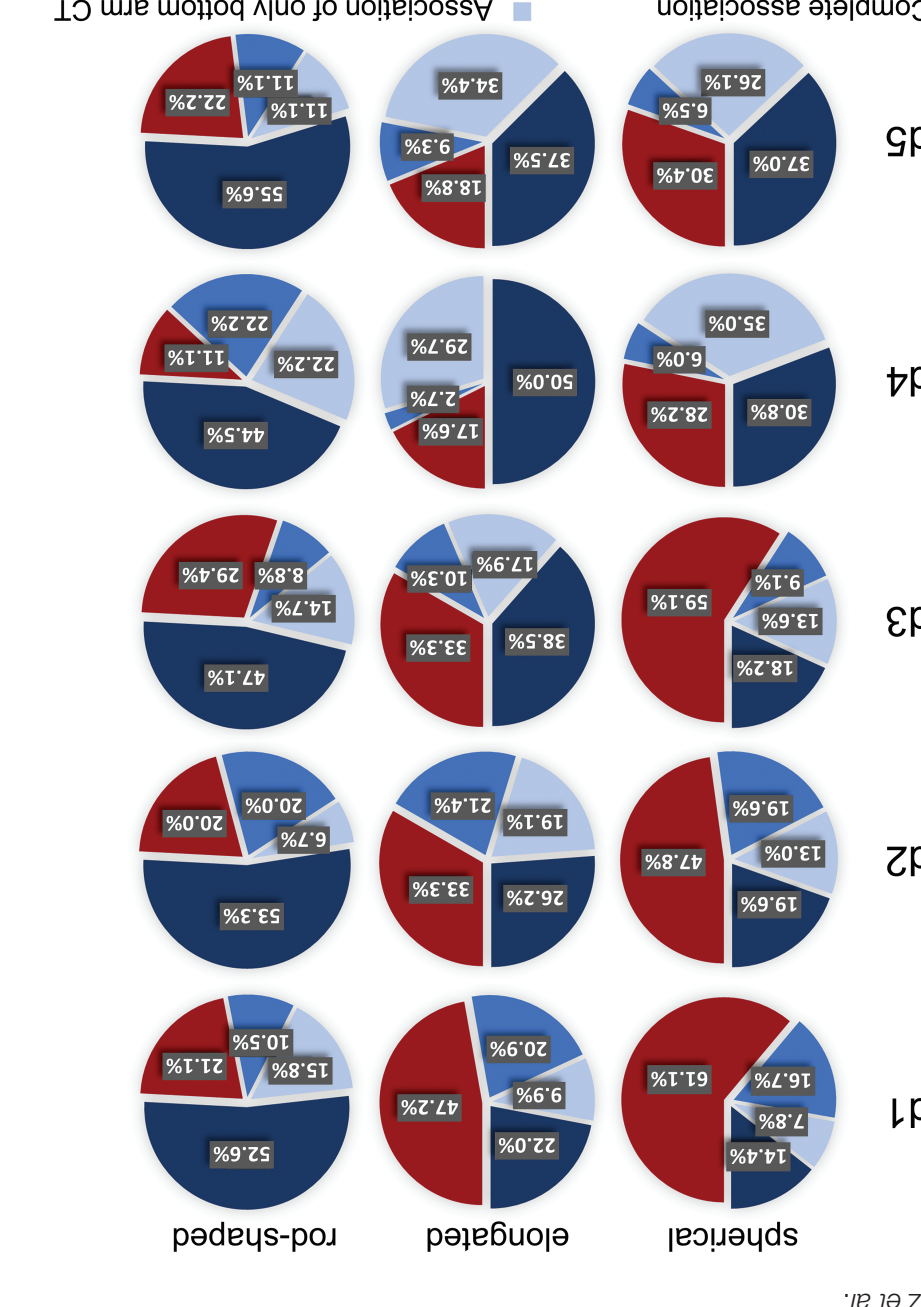
Experimentally observed associations of homologous chromosome arm territories in root nuclei of different shape (T, top arm; B, bottom arm; +, CTs associated; –, CTs separated).

The morphology of the root nuclei varied significantly from spherical via elongated to a rod-like shape. To determine whether the arrangement of CTs was related to the nuclear shape, the population of nuclei was divided into three categories based on their l/w (*x*:*y*) ratio (Fig. 3A–L; Supplementary Videos S2–S4). The percentage of nuclei with a close spatial neighbourhood of both arms of a given homologous chromosome pair was the highest in the spherical nuclei and it decreased in the elongated and rod-shaped nuclei (Fig. 4). This tendency was true for all of the *B. distachyon* chromosomes, except for Bd5. The frequency of the complete CT association of this chromosome pair in the rod-shaped nuclei was higher than in the elongated nuclei, although the difference was rather small. This might be caused by the lower number of rod-shaped nuclei analysed. In the rod-shaped nuclei, most of the homologous CTs were separated regardless of the chromosome length. Although the connection between the dominant pattern of CT distribution with the shape of a particular nucleus appeared to be easy to notice, this hypothesis could not be proved by the χ^2^ test of independence.

### Chromosome territories in spherical root nuclei associate more often than randomly

**Fig. 5.**
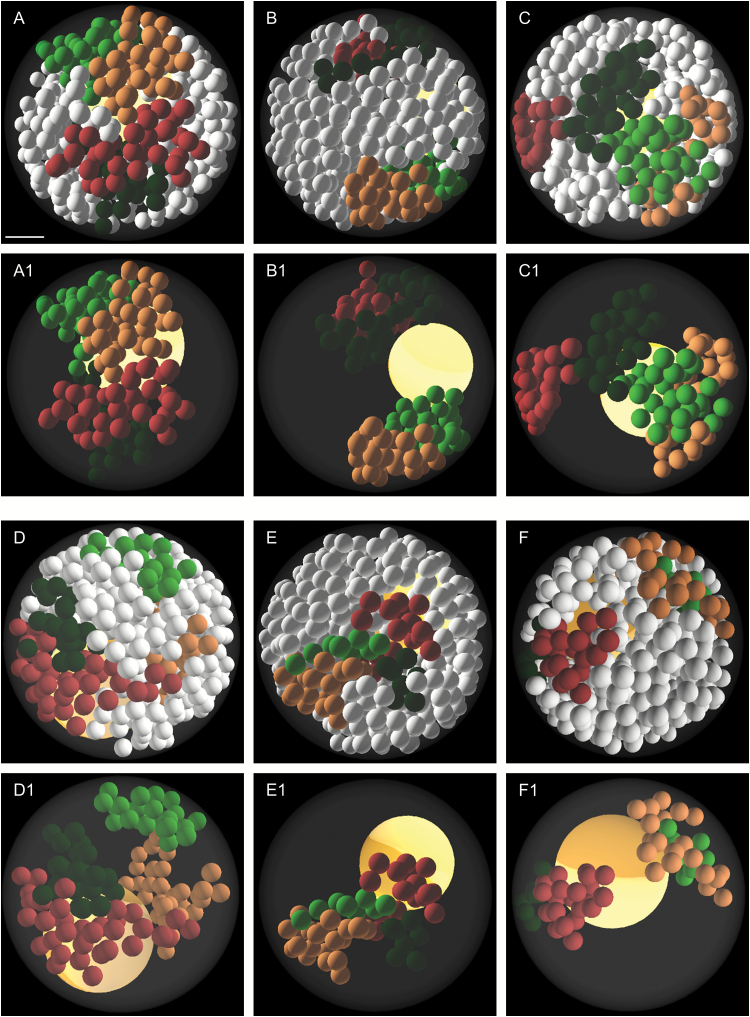
Exemplary visualization of nuclei modelled using ChroTeMo. (A, B) Nuclei with a complete association (A) or separation (B) of Bd1 chromosome arm territories. (C) Nucleus with a Bd2 top arm CT association. (D) Nucleus with a Bd3 bottom arm CT association. (E, F) Nuclei with a complete association (E) or separation (F) of Bd5 chromosome arm territories. In images (A–F), chromosomes other than the analysed pair are coloured in white. In images (A1–F1), chromosomes other than the analysed pair are transparent. One of the homologues in each image is coloured with darker shades of green and red, and the other one with lighter shades.The nucleolus is visible as a yellow sphere inside the nucleus. Scale bar=1 µm.

The theoretical frequencies of the particular types of CT associations that assumed an entirely random distribution of the chromosomes in the interphase nucleus were scored based on the simulation results that were computed by the ChroTeMo. Although the simulation of the 3-D nucleus architecture has already been performed for human nuclei and *A. thaliana* (Kreth *et al.*, 2004; Pecinka *et al.*, 2004), the software that is presented in the available publications is not publicly accessible. Thus, we decided to develop our own software, which will allow several parameters that apparently were not taken into account in the previous works to be adjusted, such as the position and size of the nucleolus (Tkacz *et al*., 2016). Our simulation also takes into account the number of chromosomes, their total length, and the position of the centromere. This script reflects the process of chromatin decondensation after mitosis in a nucleus that is represented as a sphere. As the functionality of the model is broadened, other shapes of nuclei will become available for analysis; however, at this stage of our research, only the CT distribution of spherical nuclei was compared with theoretical results. CT colouring in the model that was visualized in the ChroTeVi was analogical to the fluorescent labels that were used in the FISH experiments (green, top arms; red, bottom arms). Four types of CT distribution patterns that corresponded to the experimental observations were also noticed: the complete association of the homologues (Fig. 5A, E), the association of top (Fig. 5C) or bottom (Fig. 5D) arms, and the complete separation of CTs (Fig. 5B, F). The only difference was the presence of one additional type of CT distribution, which is the association between the top arm of one homologue with the bottom arm of another. This pattern has never been observed in our FISH experiments.

The most frequent arrangement of homologous chromosomes in the simulated nuclei was the complete separation of CTs. The only exception was Bd1, as its rendered homologues were most often associated. However, the percentage of both a complete association and a complete separation of CTs was very similar in the case of Bd1 (28.6% and 27.6%, respectively). Regardless of the chromosome pair, the observed frequency of CT separation in the nuclei after FISH was always lower and the percentage of homologue associations was higher when compared with the simulated results. To test whether these differences were statistically significant, a comparison between the experimental and simulated results was performed using the χ^2^ test of goodness of fit and was confirmed with a high confidence level (Table 2). It was shown that the observed association frequency was higher than expected for all of chromosome pairs if the simulated CT distribution was random.

**Table 2.** Comparison of the experimental and theoretical association frequencies of homologous chromosome arm territories in *B. distachyon* spherical root nuclei

Chromosome pair	Experimental frequency of the association (%)					Theoretical frequency of the association (%)				
	*n*	T+B+	T+B–	T–B+	T–B–	*n*	T+B+	T+B–	T–B+	T–B–
Bd1	90	61.1	16.7	7.8	14.4	115	28.6	17.1	26.7	27.6
Bd2	46	47.8	19.6	13.0	19.6		21.4	21.4	20.4	36.8
Bd3	44	59.1	9.1	13.6	18.2		28.7	13.9	20.4	37.0
Bd4	117	28.2	6.0	35.0	30.8		17.8	10.2	21.5	50.5
Bd5	46	30.4	6.5	26.1	37.0		11.4	7.5	23.6	57.5

T, top arm; B, bottom arm; +, CTs associated; –, CTs separated; *n*, number of nuclei analysed for each chromosome pair.

The χ^2^ test showed significant differences between the experimental and simulated results at the level *P*<0.001.

## Discussion

The chromosome painting approach that was used in this study is one of the most effective cytological methods for investigating the nuclear architecture. However, due to the unfavourable organization of repetitive sequences in the plant nuclear genome and the nearly homogenous distribution of dispersed repeats along all chromosomes, the development of chromosome paints in plants was not successful for a long time (Schubert *et al.*, 2001). The use of pools of low-copy BAC clones is one of the possibilities that can be used to deal with this problem and to analyse the internal nuclear architecture, as was demonstrated for *A. thaliana* (Pecinka *et al.*, 2004), *A. lyrata* (Berr *et al.*, 2006), and *B. distachyon* (Idziak *et al.*, 2011). Two other chromosome painting methods were recently established for *Cucumis sativus*. In one of them, pools of single-copy genes were used as probes in FISH experiments (Lou *et al.*, 2014), while the other one was based on synthetic oligonucleotides (Han *et al.*, 2015). It was shown that CT visualization using these approaches was also possible, although a complex analysis of *Cucumis* chromosome behaviour in interphase nuclei has not yet been described.

In our study, we confirmed that the interphase chromosomes of *B. distachyon* are organized in discrete CTs, as was first mentioned in this plant by Idziak *et al.* (2011). Here, we also noticed that the arm territories of the individual homologues do not overlap with the adjacent ones or that this process is limited to the areas at the borders of the territories. Such chromosome behaviour at interphase has also been described in animals and in *A. thaliana* (Pecinka *et al.*, 2004; Branco and Pombo, 2006; Cremer *et al.*, 2006; Schubert *et al.*, 2012). The territories of particular chromosomes were usually located in close proximity to the nuclear envelope, which was probably related to the low number of *B. distachyon* chromosome pairs. This feature was probably the reason for the lack of any specific radial distribution of chromosomes in the nuclei of the analysed species which is often found in animals (Croft *et al.*, 1999; Cremer *et al.*, 2001; Mayer *et al.*, 2005; Federico *et al.*, 2006; Neusser *et al.*, 2007). Thus, our attention was primarily drawn to the side-by-side arrangement of the chromosomes, in particular, to the association of the homologues. It was revealed that several factors have an influence on the *B. distachyon* nuclear architecture, such as the size of a given chromosome, its morphology, and the shape of the nucleus.

The limited amount of space within the interphase nucleus and the fact that *B. distachyon* only has five chromosome pairs are probably the main reasons why the association of homologues along their entire length was the most frequently observed type of arrangement in the case of the three longest chromosomes Bd1–Bd3. Conversely, the homologues that belong to the smaller chromosome pairs (Bd4 and Bd5) were more often separated from each other. It is to be expected that the shorter the chromosome, the higher the likelihood of the occurrence of a separated homologue.

The relationship between the CT arrangement and the morphology of a given chromosome is particularly clearly visible in the analysis of the acrocentric chromosome distribution. As one might expect, the frequency of a Bd4 and Bd5 bottom arm-only association was much higher than the occurrence of the reverse arrangement, namely the association of the homologous top arm CTs. This correlation is also observed to some extent when comparing Bd2 and Bd3. The centromere position, which determines the ratio of the chromosome arms, is not identical in both chromosomes despite their very similar length (59Mb and 60Mb, respectively). The centromere in Bd2 is more centrally located (~29Mb from the beginning of the short arm), thus its arms are of a similar length, while the centromere position in Bd3 (~25Mb) distinguishes the short and long arm more clearly. Consequently, the frequency of homologue single arm-only associations in the case of these two pairs of chromosomes most probably reflected the difference in chromosome morphology, as the top arm association of Bd2 CTs was observed more often than in Bd3. However, chromosome morphology may not be the only reason for the prevalence of a particular type of CT arrangement, as such a diversification was also observed in Bd1. The bottom arm-only association was less common, despite the almost equal chromosome arm lengths of Bd1.

Another factor that may have an impact on the prevalent type of CT arrangement in *B. distachyon* roots, although not confirmed statistically, may be the shape of the analysed nucleus. It was shown that the percentage of completely associated CTs was the highest in the spherical nuclei and that this was negatively correlated with the elongation of the nuclei. Such a correlation was also described for the nuclei of *A. thaliana* roots and leaves (Pecinka *et al.*, 2004).

It is worth noting that all of the described elements that have a direct influence on the nuclear architecture in *B. distachyon* are connected with steric constraints that are due to the limitation of the available space inside the nucleus. Such constraints are believed to play a major role in directing the overall chromatin organization in the nucleus, as was demonstrated using a new labelling technique that involves photoactivation that permits the analysis of the interphase chromosome structure and dynamics in living cells, which has been applied in studies on humans (Muller *et al.*, 2010). It was shown that the bulk of chromosome volume and shape is established rapidly at the onset of the G_1_ phase, within only an hour after mitosis. After this interval, chromosome morphology and size displayed only minor changes. These data support the view that chromosome architecture is an outcome of the subtle balance between chromosome decondensation and the constraints that are imposed by the nuclear envelope, other nuclear compartments, and the surrounding chromosomes. Similarly, low chromatin motility was observed in the interphase nuclei of the stamen hairs of *Tradescantia paludosa*, in spite of the fact that they displayed some flexibility (Schubert *et al.*, 2014). The results of a computer simulation of *A. thaliana* chromosomes, which showed that the organization and position of chromosomes is affected by the nuclear space, further substantiates this hypothesis (de Nooijer *et al.*, 2009).

The population of nuclei that had been isolated from *B. distachyon* roots contained a mixed proportion of meristematic and differentiated cells. Some of these were also endoreduplicated, as they could easily be distinguished in the preparations based on their size. Their presence in the roots of the analysed species was also previously confirmed using digital image cytometry (Idziak *et al.*, 2015). Nevertheless, no changes in the CT organization in the endopolyploid cells was observed and the maximal number of homologue territories was never greater than two, thus suggesting that the duplicated chromatids are retained together even after several rounds of endoreduplication. This observation is in accordance with our results of centromere and telomere mapping in *Brachypodium* species, in which the number of analysed sequences did not significantly increase in the endoreduplicated root nuclei (Idziak *et al.*, 2015). The lack of major diversification in terms of chromosome spatial arrangement between meristematic and differentiated cells was also described by Berr and Schubert (2007) for *A. thaliana*, which indicates no substantial reorganization of the nuclear architecture in plants during differentiation and after the first endopolyploidization steps (Schubert *et al.*, 2012).

The comparison of the results obtained by FISH and from the simulation of CT arrangement using ChroTeMo revealed that homologous arm CTs associate more often than if they were randomly distributed inside of the interphase nucleus. Interestingly, we did not observe a significant increase in the frequency of the association of the Bd5 top arm. This might have been expected due to the presence of 35S rDNA loci in the top arm of this chromosome pair (Draper *et al.*, 2001) and the formation of a joint nucleolus in most of the nuclei. It is probable that the function of rRNA genes is reflected in the association of whole Bd5 CTs, which occurred more often in the actual material than predicted by the model. We also cannot rule out that the frequency of Bd5 associations was underestimated due to the elimination of BAC clones that contained 35S rDNA from the painting probes, which might have caused some technical issues.

The preferential association of homologous chromosomes was reported in some eukaryotic species. Such chromatin organization was described in the somatic cells of *Drosophila melanogaster* (Csink and Henikoff, 1998; Fung *et al.*, 1998) or in murine haematopoietic cells (Kosak *et al.*, 2007; Rajapakse *et al.*, 2009). In contrast, there was no preferential association of homologues in human cancer cells (Heride *et al.*, 2010) and in lymphocytes of mice (Caddle *et al.*, 2007). In *A. thaliana*, only the NOR-bearing chromosomes 2 and 4 associate more often, while the other chromosome pairs are distributed randomly (Pecinka *et al.*, 2004). The closely related *A. lyrata* also retains this trend, despite having a larger nuclear genome and a more diversified karyotype (Berr *et al.*, 2006). However, *B. distachyon*, as a representative of monocotyledonous plants, may present a nuclear organization that is different from that of dicotyledonous *A. thaliana*. Some feedback for this hypothesis can be obtained from the studies of the wheat–barley substitution lines that contained a pair of barley chromosomes, where the barley homologues were often associated in the pre-meiotic and tapetum nuclei (Aragon-Alcaide *et al.*, 1997).

In most of the *B. distachyon* root nuclei, a Rabl-like configuration with the centromeric and telomeric sequences localized at the opposite poles of the nuclei was recently described by our group (Idziak *et al.*, 2015). The presence of such a nuclear architecture may be linked to the prevalent association of homologues to some extent as the connection between the number of nuclei with the centromere and telomere polarization and the nuclear shape was also observed. It is worth noting that the frequency of both the occurrence of a Rabl configuration and a homologous chromosome association was negatively correlated with the elongation of the nuclei. Nevertheless, at this stage of our research, we cannot determine if one of these phenomena is the cause and the other the reason or whether they are both a consequence of some other factors that determine the nuclear architecture.

The association of entire CTs may not indicate the pairing of the homologues along their entire length. The mapping of unique ~100kb BAC clones of *A. thaliana* showed that the frequency of the association of particular chromosome regions was 7–10 times lower that the association of entire CTs (Pecinka *et al.*, 2004). There is also a very compelling assumption that homologous chromosome association is based on the interaction between the heterochromatic regions of chromosomes rather than the euchromatin (Pecinka *et al.*, 2005; Jovtchev *et al.*, 2008).

Despite all of the data gathered so far, we are still along way from understanding how the internal organization of the CTs inside the nucleus is established and regulated. It is believed that chromatin distribution in the interphase nucleus has an impact on many nuclear processes, such as DNA replication, transcription, or repair, and that therefore the association of homologous chromosomes is necessary for the cell, at least temporarily (Heard and Bickmore, 2007; Misteli, 2007; Mekhail and Moazed, 2010). Depending on the current needs of the cell, the tissue being analysed, or the developmental stage, the architecture of the nucleus may be reorganized (Chakalova *et al.*, 2005; Misteli and Soutoglou, 2009; Papantonis and Cook, 2010). Our studies on the nucleus organization in *B. distachyon* are likely to constitute a suitable starting point to understand these changes; for example, in response to environmental conditions or to biotic and abiotic stresses.

## Supplementary data

Supplementary data are available at *JXB* online.

Figure S1. Volumes of the nucleoli in the root cell nuclei of *B. distachyon*.

Figure S2. Size of the nucleoli isolated from *B. distachyon* roots.

Table S1. Characteristics of BAC clones used for the chromosome painting of Bd1.

Table S2. Characteristics of BAC clones used for the chromosome painting of Bd2.

Table S3. Characteristics of BAC clones used for the chromosome painting of Bd3.

Table S4. Characteristics of BAC clones used for the chromosome painting of Bd4.

Table S5. Characteristics of BAC clones used for the chromosome painting of Bd5.

Video S1. Models of the nuclei of *B. distachyon* root cells with visible nucleoli that are presented in Supplementary Fig. S2A and B.

Video S2. Arrangement of Bd4 homologous CTs in the spherical nuclei of *B. distachyon* root cells: (A) complete association of homologues that is presented in Fig. 3A; (B) association of only the top arm CTs that is presented in Fig. 3B; (C) association of only the bottom arm CTs that is presented in Fig. 3C; (D) complete separation of the homologues that is presented in Fig. 3D.

Video S3. Arrangement of Bd4 homologous CTs in the elongated nuclei of *B. distachyon* root cells: (A) complete association of the homologues that is presented in Fig. 3E; (B) association of only the top arm CTs that is presented in Fig. 3F; (C) association of only the bottom arm CTs that is presented in Fig. 3G; (D) complete separation of the homologues that is presented in Fig. 3H.

Video S4. Arrangement of the Bd4 homologous CTs in the rod-shaped nuclei of *B. distachyon* root cells: (A) complete association of the homologues that is presented in Fig. 3I; (B) association of only the top arm CTs that is presented in Fig. 3J; (C) association of only the bottom arm CTs that is presented in Fig. 3K; (D) complete separation of the homologues that is presented in Fig. 3L.

Supplementary Data

## References

[CIT0001] Albiez H Cremer M Tiberi C 2006 Chromatin domains and the interchromatin compartment form structurally defined and functionally interacting nuclear networks. Chromosome Research 14, 707–733. 17115328 10.1007/s10577-006-1086-x

[CIT0002] Aragon-Alcaide L Reader S Beven A Shaw P Miller T Moore G 1997 Association of homologous chromosomes during floral development. Current Biology 7, 905–908. 9382806 10.1016/s0960-9822(06)00383-6

[CIT0003] Bennett MD Leitch IJ 2005 Nuclear DNA amounts in angiosperms: progress, problems and prospects. Annals of Botany 95, 45–90. 15596457 10.1093/aob/mci003 PMC4246708

[CIT0004] Berr A Pecinka A Meister A Kreth G Fuchs J Blattner FR Lysak MA Schubert I 2006 Chromosome arrangement and nuclear architecture but not centromeric sequences are conserved between *Arabidopsis thaliana* and *Arabidopsis lyrata* . The Plant Journal 48, 771–783. 17118036 10.1111/j.1365-313X.2006.02912.x

[CIT0005] Berr A Schubert I 2007 Interphase chromosome arrangement in *Arabidopsis thaliana* is similar in differentiated and meristematic tissues and shows a transient mirror symmetry after nuclear division. Genetics 176, 853–863. 17409060 10.1534/genetics.107.073270 PMC1894613

[CIT0006] Betekhtin A Jenkins G Hasterok R 2014 Reconstructing the evolution of *Brachypodium* genomes using comparative chromosome painting. PLoS One 9, e115108. 25493646 10.1371/journal.pone.0115108 PMC4262448

[CIT0007] Bolzer A Kreth G Solovei I 2005 Three-dimensional maps of all chromosomes in human male fibroblast nuclei and prometaphase rosettes. PLoS Biology 3, e157. 15839726 10.1371/journal.pbio.0030157 PMC1084335

[CIT0008] Boyle S Gilchrist S Bridger JM Mahy NL Ellis JA Bickmore WA 2001 The spatial organization of human chromosomes within the nuclei of normal and emerin-mutant cells. Human Molecular Genetics 10, 211–219. 11159939 10.1093/hmg/10.3.211

[CIT0009] Branco MR Pombo A 2006 Intermingling of chromosome territories in interphase suggests role in translocations and transcription-dependent associations. PLoS Biology 4, e138. 16623600 10.1371/journal.pbio.0040138 PMC1440941

[CIT0010] Bridger JM Boyle S Kill IR Bickmore WA 2000 Re-modelling of nuclear architecture in quiescent and senescent human fibroblasts. Current Biology 10, 149–152. 10679329 10.1016/s0960-9822(00)00312-2

[CIT0011] Caddle LB Grant JL Szatkiewicz J Van Hase J Shirley B-J Bewersdorf J Cremer C Arneodo A Khalil A Mills KD 2007 Chromosome neighborhood composition determines translocation outcomes after exposure to high-dose radiation in primary cells. Chromosome Research 15, 1061–1073. 18060570 10.1007/s10577-007-1181-7

[CIT0012] Catalan P Muller J Hasterok R Jenkins G Mur LA Langdon T Betekhtin A Siwinska D Pimentel M Lopez-Alvarez D 2012 Evolution and taxonomic split of the model grass *Brachypodium distachyon* . Annals of Botany 109, 385–405. 22213013 10.1093/aob/mcr294 PMC3268539

[CIT0013] Chakalova L Carter D Debrand E Goyenechea B Horton A Miles J Osborne C Fraser P 2005 Developmental regulation of the beta-globin gene locus. Progress in Molecular and Subcellular Biology 38, 183–206. 15881896 10.1007/3-540-27310-7_8

[CIT0014] Chandley AC Speed RM Leitch AR 1996 Different distributions of homologous chromosomes in adult human Sertoli cells and in lymphocytes signify nuclear differentiation. Journal of Cell Science 109, 773–776. 8718668 10.1242/jcs.109.4.773

[CIT0015] Cremer M von Hase J Volm T Brero A Kreth G Walter J Fischer C Solovei I Cremer C Cremer T 2001 Non-random radial higher-order chromatin arrangements in nuclei of diploid human cells. Chromosome Research 9, 541–567. 11721953 10.1023/a:1012495201697

[CIT0016] Cremer T Cremer C Baumann H Luedtke EK Sperling K Teuber V Zorn C 1982 Rabl’s model of the interphase chromosome arrangement tested in Chinese hamster cells by premature chromosome condensation and laser-UV-microbeam experiments. Human Genetics 60, 46–56. 7076247 10.1007/BF00281263

[CIT0017] Cremer T Cremer M Dietzel S Muller S Solovei I Fakan S 2006 Chromosome territories—a functional nuclear landscape. Current Opinion in Cell Biology 18, 307–316. 16687245 10.1016/j.ceb.2006.04.007

[CIT0018] Croft JA Bridger JM Boyle S Perry P Teague P Bickmore WA 1999 Differences in the localization and morphology of chromosomes in the human nucleus. Journal of Cell Biology 145, 1119–1131. 10366586 10.1083/jcb.145.6.1119 PMC2133153

[CIT0019] Csink AK Henikoff S 1998 Large-scale chromosomal movements during interphase progression in *Drosophila* . Journal of Cell Biology 143, 13–22. 9763417 10.1083/jcb.143.1.13 PMC2132807

[CIT0020] de Nooijer S Wellink J Mulder B Bisseling T 2009 Non-specific interactions are sufficient to explain the position of heterochromatic chromocenters and nucleoli in interphase nuclei. Nucleic Acids Research 37, 3558–3568. 19359359 10.1093/nar/gkp219 PMC2699506

[CIT0021] Dolezel J Binaroca P Lucretti S 1989 Analysis of nuclear DNA content in plant cells by flow cytometry. Biologia Plantarum 31, 113–120.

[CIT0022] Dong F Jiang J 1998 Non-Rabl patterns of centromere and telomere distribution in the interphase nuclei of plant cells. Chromosome Research 6, 551–558. 9886774 10.1023/a:1009280425125

[CIT0023] Draper J Mur LA Jenkins G Ghosh-Biswas GC Bablak P Hasterok R Routledge AP 2001 *Brachypodium distachyon*. A new model system for functional genomics in grasses. Plant Physiology 127, 1539–1555. 11743099 PMC133562

[CIT0024] Febrer M Goicoechea JL Wright J 2010 An integrated physical, genetic and cytogenetic map of *Brachypodium distachyon*, a model system for grass research. PLoS One 5, e13461. 20976139 10.1371/journal.pone.0013461 PMC2956642

[CIT0025] Federico C Scavo C Cantarella CD Motta S Saccone S Bernardi G 2006 Gene-rich and gene-poor chromosomal regions have different locations in the interphase nuclei of cold-blooded vertebrates. Chromosoma 115, 123–128. 16404627 10.1007/s00412-005-0039-z

[CIT0026] Funabiki H Hagan I Uzawa S Yanagida M 1993 Cell cycle-dependent specific positioning and clustering of centromeres and telomeres in fission yeast. Journal of Cell Biology 121, 961–976. 8388878 10.1083/jcb.121.5.961 PMC2119680

[CIT0027] Fung JC Marshall WF Dernburg A Agard DA Sedat JW 1998 Homologous chromosome pairing in *Drosophila melanogaster* proceeds through multiple independent initiations. Journal of Cell Biology 141, 5–20. 9531544 10.1083/jcb.141.1.5 PMC2132734

[CIT0028] Fussell CP 1992 Rabl distribution of interphase and prophase telomeres in *Allium cepa* not altered by colchicine and/or ultracentrifugation. American Journal of Botany 79, 771–777.

[CIT0029] Garvin D Gu Y Hasterok R Hazen S Jenkins G Mockler T Mur L Vogel J 2008 Development of genetic and genomic research resources for *Brachypodium distachyon*, a new model system for grass crop research. Crop Science 48, S69–S84.

[CIT0030] Habermann FA Cremer M Walter J Kreth G von Hase J Bauer K Wienberg J Cremer C Cremer T Solovei I 2001 Arrangements of macro- and microchromosomes in chicken cells. Chromosome Research 9, 569–584. 11721954 10.1023/a:1012447318535

[CIT0031] Han Y Zhang T Thammapichai P Weng Y Jiang J 2015 Chromosome-specific painting in *Cucumis* species using bulked oligonucleotides. Genetics 200, 771–779. 25971668 10.1534/genetics.115.177642 PMC4512542

[CIT0032] Harrison GE Heslop-Harrison JS 1995 Centromeric repetitive DNA sequences in the genus *Brassica* . Theoretical and Applied Genetics 90, 157–165. 24173886 10.1007/BF00222197

[CIT0033] Heard E Bickmore W 2007 The ins and outs of gene regulation and chromosome territory organisation. Current Opinion in Cell Biology 19, 311–316. 17467967 10.1016/j.ceb.2007.04.016

[CIT0034] Heride C Ricoul M Kieu K von Hase J Guillemot V Cremer C Dubrana K Sabatier L 2010 Distance between homologous chromosomes results from chromosome positioning constraints. Journal of Cell Science 123, 4063–4075. 21084563 10.1242/jcs.066498

[CIT0035] Hochstrasser M Mathog D Gruenbaum Y Saumweber H Sedat JW 1986 Spatial organization of chromosomes in the salivary gland nuclei of *Drosophila melanogaster* . Journal of Cell Biology 102, 112–123. 3079766 10.1083/jcb.102.1.112 PMC2114037

[CIT0036] Idziak D Betekhtin A Wolny E Lesniewska K Wright J Febrer M Bevan MW Jenkins G Hasterok R 2011 Painting the chromosomes of *Brachypodium*: current status and future prospects. Chromosoma 120, 469–479. 21667205 10.1007/s00412-011-0326-9 PMC3174371

[CIT0037] Idziak D Robaszkiewicz E Hasterok R 2015 Spatial distribution of centromeres and telomeres at interphase varies among *Brachypodium* species. Journal of Experimental Botany 66, 6623–6634. 26208647 10.1093/jxb/erv369 PMC4623680

[CIT0038] International Brachypodium Initiative 2010 Genome sequencing and analysis of the model grass *Brachypodium distachyon* . Nature 463, 763–768. 20148030 10.1038/nature08747

[CIT0039] Jasencakova Z Meister A Schubert I 2001 Chromatin organization and its relation to replication and histone acetylation during the cell cycle in barley. Chromosoma 110, 83–92. 11453558 10.1007/s004120100132

[CIT0040] Jenkins G Hasterok R 2007 BAC ‘landing’ on chromosomes of *Brachypodium distachyon* for comparative genome alignment. Nature Protocols 2, 88–98. 17401342 10.1038/nprot.2006.490

[CIT0041] Jin Q Trelles-Sticken E Scherthan H Loidl J 1998 Yeast nuclei display prominent centromere clustering that is reduced in nondividing cells and in meiotic prophase. Journal of Cell Biology 141, 21–29. 9531545 10.1083/jcb.141.1.21 PMC2132713

[CIT0042] Jovtchev G Watanabe K Pecinka A Rosin FM Mette MF Lam E Schubert I 2008 Size and number of tandem repeat arrays can determine somatic homologous pairing of transgene loci mediated by epigenetic modifications in *Arabidopsis thaliana* nuclei. Chromosoma 117, 267–276. 18200447 10.1007/s00412-007-0146-0

[CIT0043] Kamm A Galasso I Schmidt T Heslop-Harrison JS 1995 Analysis of a repetitive DNA family from *Arabidopsis arenosa* and relationships between *Arabidopsis* species. Plant Molecular Biology 27, 853–862. 7766876 10.1007/BF00037014

[CIT0044] Koehler D Zakhartchenko V Froenicke L Stone G Stanyon R Wolf E Cremer T Brero A 2009 Changes of higher order chromatin arrangements during major genome activation in bovine preimplantation embryos. Experimental Cell Research 315, 2053–2063. 19254712 10.1016/j.yexcr.2009.02.016

[CIT0045] Kosak ST Scalzo D Alworth SV Li F Palmer S Enver T Lee JS Groudine M 2007 Coordinate gene regulation during hematopoiesis is related to genomic organization. PLoS Biology 5, e309. 18031200 10.1371/journal.pbio.0050309 PMC2080650

[CIT0046] Kreth G Finsterle J von Hase J Cremer M Cremer C 2004 Radial arrangement of chromosome territories in human cell nuclei: a computer model approach based on gene density indicates a probabilistic global positioning code. Biophysical Journal 86, 2803–2812. 15111398 10.1016/S0006-3495(04)74333-7 PMC1304150

[CIT0047] Lichter P Cremer T Borden J Manuelidis L Ward DC 1988 Delineation of individual human chromosomes in metaphase and interphase cells by *in situ* suppression hybridization using recombinant DNA libraries. Human Genetics 80, 224–234. 3192212 10.1007/BF01790090

[CIT0048] Lou Q Zhang Y He Y Li J Jia L Cheng C Guan W Yang S Chen J 2014 Single-copy gene-based chromosome painting in cucumber and its application for chromosome rearrangement analysis in *Cucumis* . The Plant Journal 78, 169–179. 24635663 10.1111/tpj.12453

[CIT0049] Mahy NL Perry PE Gilchrist S Baldock RA Bickmore WA 2002 Spatial organization of active and inactive genes and noncoding DNA within chromosome territories. Journal of Cell Biology 157, 579–589. 11994314 10.1083/jcb.200111071 PMC2173868

[CIT0050] Manuelidis L 1985 Individual interphase chromosome domains revealed by *in situ* hybridization. Human Genetics 71, 288–293. 3908288 10.1007/BF00388453

[CIT0051] Mayer R Brero A von Hase J Schroeder T Cremer T Dietzel S 2005 Common themes and cell type specific variations of higher order chromatin arrangements in the mouse. BMC Cell Biology 6, 44. 16336643 10.1186/1471-2121-6-44 PMC1325247

[CIT0052] Mekhail K Moazed D 2010 The nuclear envelope in genome organization, expression and stability. Nature Reviews *Molecular Cell Biology* 11, 317–328. 20414256 10.1038/nrm2894 PMC3246372

[CIT0053] Misteli T 2007 Beyond the sequence: cellular organization of genome function. Cell 128, 787–800. 17320514 10.1016/j.cell.2007.01.028

[CIT0054] Misteli T Soutoglou E 2009 The emerging role of nuclear architecture in DNA repair and genome maintenance. Nature Reviews *Molecular Cell Biology* 10, 243–254. 19277046 10.1038/nrm2651 PMC3478884

[CIT0055] Muller I Boyle S Singer RH Bickmore WA Chubb JR 2010 Stable morphology, but dynamic internal reorganisation, of interphase human chromosomes in living cells. PLoS One 5, e11560. 20644634 10.1371/journal.pone.0011560 PMC2903487

[CIT0056] Nagele RG Freeman T McMorrow L Thomson Z Kitson-Wind K Lee H 1999 Chromosomes exhibit preferential positioning in nuclei of quiescent human cells. Journal of Cell Science 112, 525–535. 9914164 10.1242/jcs.112.4.525

[CIT0057] Neusser M Schubel V Koch A Cremer T Muller S 2007 Evolutionarily conserved, cell type and species-specific higher order chromatin arrangements in interphase nuclei of primates. Chromosoma 116, 307–320. 17318634 10.1007/s00412-007-0099-3

[CIT0058] Papantonis A Cook PR 2010 Genome architecture and the role of transcription. Current Opinion in Cell Biology 22, 271–276. 20356724 10.1016/j.ceb.2010.03.004 PMC2884177

[CIT0059] Parada LA McQueen PG Misteli T 2004 Tissue-specific spatial organization of genomes. Genome Biology 5, R44. 15239829 10.1186/gb-2004-5-7-r44 PMC463291

[CIT0060] Pecinka A Kato N Meister A Probst AV Schubert I Lam E 2005 Tandem repetitive transgenes and fluorescent chromatin tags alter local interphase chromosome arrangement in *Arabidopsis thaliana* . Journal of Cell Science 118, 3751–3758. 16076901 10.1242/jcs.02498

[CIT0061] Pecinka A Schubert V Meister A Kreth G Klatte M Lysak MA Fuchs J Schubert I 2004 Chromosome territory arrangement and homologous pairing in nuclei of *Arabidopsis thaliana* are predominantly random except for NOR-bearing chromosomes. Chromosoma 113, 258–269. 15480725 10.1007/s00412-004-0316-2

[CIT0062] Pinkel D Landegent J Collins C Fuscoe J Segraves R Lucas J Gray J 1988 Fluorescence *in situ* hybridization with human chromosome-specific libraries: detection of trisomy 21 and translocations of chromosome 4. Proceedings of the National Academy of Sciences, USA 85, 9138–9142. 10.1073/pnas.85.23.9138 PMC282679 2973607

[CIT0063] Rabl C 1885 Uber Zelltheilung. Morphologisches Jahrbuch 10, 214–330.

[CIT0064] Rajapakse I Perlman MD Scalzo D Kooperberg C Groudine M Kosak ST 2009 The emergence of lineage-specific chromosomal topologies from coordinate gene regulation. Proceedings of the National Academy of Sciences, USA 106, 6679–6684. 10.1073/pnas.0900986106 PMC2654024 19276122

[CIT0065] Rawlins DJ Highett MI Shaw PJ 1991 Localization of telomeres in plant interphase nuclei by *in situ* hybridization and 3D confocal microscopy. Chromosoma 100, 424–431.

[CIT0066] Rothbart SB Strahl BD 2014 Interpreting the language of histone and DNA modifications. Biochimica et Biophysica Acta 1839, 627–643. 24631868 10.1016/j.bbagrm.2014.03.001 PMC4099259

[CIT0067] Schubert I Fransz PF Fuchs J de Jong JH 2001 Chromosome painting in plants. Methods in Cell Science 23, 57–69. 11741144

[CIT0068] Schubert V Berr A Meister A 2012 Interphase chromatin organisation in *Arabidopsis* nuclei: constraints versus randomness. Chromosoma 121, 369–387. 22476443 10.1007/s00412-012-0367-8

[CIT0069] Schubert V Meister A Tsujimoto H Endo TR Houben A 2011 Similar rye A and B chromosome organization in meristematic and differentiated interphase nuclei. Chromosome Research 19, 645–655. 21674259 10.1007/s10577-011-9224-5

[CIT0070] Schubert V Rudnik R Schubert I 2014 Chromatin associations in *Arabidopsis* interphase nuclei. Frontiers in Genetics 5, 389. 25431580 10.3389/fgene.2014.00389 PMC4230181

[CIT0071] Sharma SK Yamamoto M Mukai Y 2015 Immuno-cytogenetic manifestation of epigenetic chromatin modification marks in plants. Planta 241, 291–301. 25539867 10.1007/s00425-014-2233-9

[CIT0072] Sun HB Shen J Yokota H 2000 Size-dependent positioning of human chromosomes in interphase nuclei. Biophysical Journal 79, 184–190. 10866946 10.1016/S0006-3495(00)76282-5 PMC1300924

[CIT0073] Swygert SG Peterson CL 2014 Chromatin dynamics: interplay between remodeling enzymes and histone modifications. Biochimica et Biophysica Acta 1839, 728–736. 24583555 10.1016/j.bbagrm.2014.02.013 PMC4099280

[CIT0074] Tanabe H Muller S Neusser M von Hase J Calcagno E Cremer M Solovei I Cremer C Cremer T 2002 Evolutionary conservation of chromosome territory arrangements in cell nuclei from higher primates. Proceedings of the National Academy of Sciences, USA 99, 4424–4429. 10.1073/pnas.072618599 PMC123664 11930003

[CIT0074a] Tkacz MA Chromiński K Idziak-Helmcke D Robaszkiewicz E Hasterok R 2016 Chromosome territory modeller and viewer. PLoS One 11, e0160303. 10.1371/journal.pone.0160303 PMC4978479 27505434

[CIT0075] Venkatesh S Workman JL 2015 Histone exchange, chromatin structure and the regulation of transcription. Nature Reviews *Molecular Cell Biology* 16, 178–189. 25650798 10.1038/nrm3941

[CIT0076] Visser AE Jaunin F Fakan S Aten JA 2000 High resolution analysis of interphase chromosome domains. Journal of Cell Science 113, 2585–2593. 10862716 10.1242/jcs.113.14.2585

[CIT0077] Zeitz MJ Mukherjee L Bhattacharya S Xu J Berezney R 2009 A probabilistic model for the arrangement of a subset of human chromosome territories in WI38 human fibroblasts. Journal of Cellular Physiology 221, 120–129. 19507193 10.1002/jcp.21842

