# Supplementary material for: The arrangement of Brachypodium distachyon chromosomes in interphase nuclei
Source: J Exp Bot. 2016 Sep 1;67(18):5571–83. doi: 10.1093/jxb/erw325 (PMC5049400; doi:10.1093/jxb/erw325)
Supplement: Supplementary Data [file supp_67_18_5571__index.html]

The arrangement of Brachypodium distachyon chromosomes in interphase nuclei — The arrangement of Brachypodium distachyon chromosomes in interphase nuclei — Supplementary Data 

# The arrangement of *Brachypodium distachyon* chromosomes in interphase nuclei

## Supplementary Data

Data files

- Supplementary\_Figures\_S1\_S2.pdf - Supplementary Data
- Supplementary\_Tables\_S1\_S5.pdf - Supplementary Data
- Supplementary\_Video\_S1A.avi - Supplementary Data
- Supplementary\_Video\_S1B.avi - Supplementary Data
- Supplementary\_Video\_S2A.avi - Supplementary Data
- Supplementary\_Video\_S2B.avi - Supplementary Data
- Supplementary\_Video\_S2C.avi - Supplementary Data
- Supplementary\_Video\_S2D.avi - Supplementary Data
- Supplementary\_Video\_S3A.avi - Supplementary Data
- Supplementary\_Video\_S3B.avi - Supplementary Data
- Supplementary\_Video\_S3C.avi - Supplementary Data
- Supplementary\_Video\_S3D.avi - Supplementary Data
- Supplementary\_Video\_S4A.avi - Supplementary Data
- Supplementary\_Video\_S4B.avi - Supplementary Data
- Supplementary\_Video\_S4C.av - Supplementary Data
- Supplementary\_Video\_S4D.avi - Supplementary Data
