# Supplementary material for: The arrangement of Brachypodium distachyon chromosomes in interphase nuclei
Source: J Exp Bot. 2016 Sep 1;67(18):5571–83. doi: 10.1093/jxb/erw325 (PMC5049400; doi:10.1093/jxb/erw325)
Supplement: Supplementary Data [file supp_erw325_Supplementary_Figures_S1_S2.pdf]

Journal of Experimental Botany

The distribution and behaviour of *Brachypodium distachyon* chromosomes at interphase

Ewa Robaszkiewicz, Dominika Idziak-Helmcke, Magdalena Tkacz, Kornel Chromiński and Robert Hasterok

SUPPLEMENTARY DATA

Supplementary Figure S1

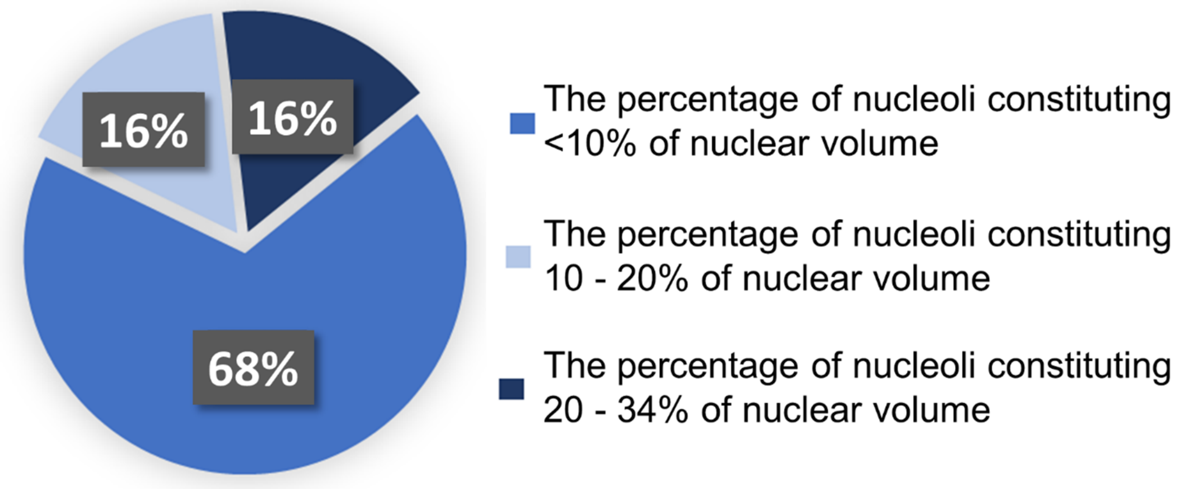

Supplementary Figure S1. Volumes of the nucleoli in the root cell nuclei of *B. distachyon*.

Supplementary Figure S2

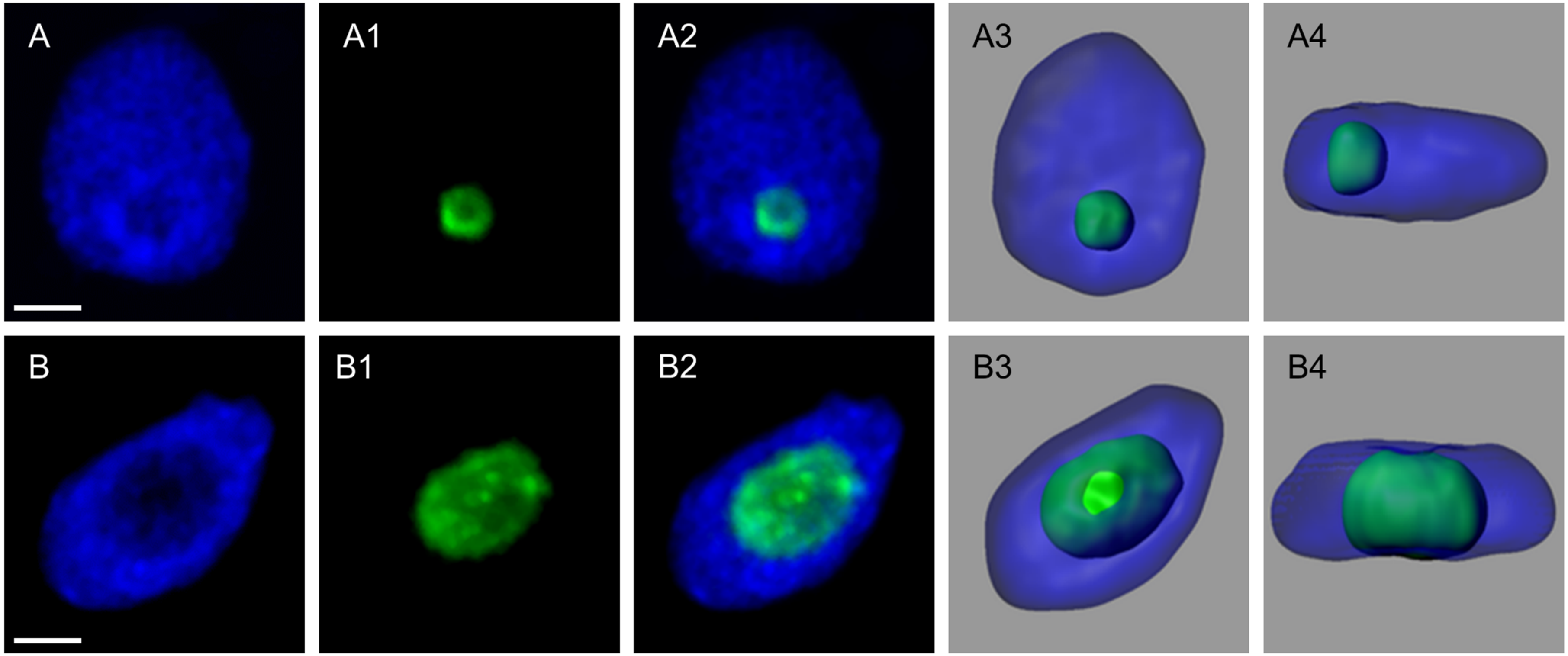

Supplementary Figure S2. Size of the nucleoli isolated from *B. distachyon* roots.

(A, B) Interphase nuclei with a similar volume (78.7 and 73.4 μm^3, respectively), counterstained with DAPI (blue fluorescence);

(A1, B1) Nucleoli with a different volume (3.8 and 23.7 μm^3, respectively) detected by immunostaining fibrillarin (green fluorescence);

(A2, B2) Superimposed images of nuclei and nucleoli;

(A3-A4, B3-B4) Models of nuclei and nucleoli prepared using Imaris Software (Bitplane) based on microscopy images and observed in different planes.

Scale bars: 2 μm.
