# Supplementary material for: The arrangement of Brachypodium distachyon chromosomes in interphase nuclei
Source: J Exp Bot. 2016 Sep 1;67(18):5571–83. doi: 10.1093/jxb/erw325 (PMC5049400; doi:10.1093/jxb/erw325)
Supplement: Supplementary Data [file supp_erw325_Supplementary_Tables_S1_S5.pdf]

Supplementary Table S1. Characteristics of BAC clones used for the chromosome painting of Bd1.

Bd1 short arm				
**No.**	**Clone ID**	**Start [bp]**	**End [bp]**	**Repeat content [%]**
1	a0035K02	147863	304506	18.31
2	b0027N17	560624	710332	6.56
3	a0037D23	1171403	1328435	13.07
4	a0012F06	1537097	1734409	7.59
5	a0032E05	1907231	2063694	11.83
6	a0008O14	2635548	2801693	8.30
7	a0021B03	3028832	3173186	6.23
8	a0004B12	3276891	3460444	5.68
9	b0044D24	3878248	4004060	13.91
10	b0003A11	4404030	4546882	30.82
11	a0032K13	5048843	5206517	26.89
12	a0017K22	5375697	5509098	17.71
13	b0037O18	6122656	6272292	19.17
14	a0022C04	6574012	6741405	30.61
15	b0040G07	7221475	7389553	30.09
16	b0013P15	7656602	7805960	29.21
17	a0013O16	7926543	8055682	39.20
18	b0030L10	8680898	8845282	10.03
19	b0012L20	8850673	9007358	14.08
20	a0015H06	9424591	9569999	14.80
21	a0007G23	9965773	10101237	15.91
22	a0032D10	10490542	10652198	11.18
23	a0003J21	10927667	11073109	16.93
24	a0023P13	11505702	11632287	22.48
25	a0032F08	12115980	12241228	17.12
26	a0024M10	12444261	12575731	23.81
27	a0027D04	12706461	12847057	22.87
28	b0019G20	13362834	13517753	21.02
29	b0023C02	13999817	14137163	13.45
30	b0001G04	14561692	14709543	15.53

Bd1 short arm				
No.	Clone ID	Start [bp]	End [bp]	Repeat content [%]
31	a0020A04	15092918	15238493	0.00
32	b0023O18	15449374	15577903	0.00
33	a0003N21	16107590	16251236	10.80
34	a0002N01	16344468	16496573	11.12
35	a0009N18	17150298	17335777	0.00
36	a0014L23	17404191	17543242	7.86
37	b0018P22	18190466	18326563	23.15
38	a0017E13	18574112	18708723	0.00
39	a0010I03	19198770	19342731	7.06
40	a0007L04	19364297	19511432	8.67
41	b0002O16	20013520	20160236	0.00
42	a0024N14	20488400	20631457	19.18
43	a0027K03	21168673	21307307	0.00
44	a0010K04	21496092	21643627	19.83
45	a0011I01	21907910	22040598	0.00
46	a0018B03	22412015	22565632	32.71
47	b0022H13	23114454	23242441	0.00
48	a0023E14	23230575	23392276	21.52
49	a0043B06	24028749	24191469	22.76
50	a0042C21	24228323	24375228	16.48
51	a0026E19	25017625	25161139	14.31
52	a0046B12	25556278	25718695	17.60
53	a0018O15	25727688	25878318	13.18
54	b0028A06	26442023	26591576	16.02
55	b0002C04	27060765	27214938	27.84
56	a0006K13	27522409	27682274	23.09
57	a0044I06	28135872	28292480	21.81
58	a0043P17	28526824	28683718	21.98
59	a0029A09	28940084	29079192	22.18
60	a0032C01	29475135	29677346	31.42
61	a0002G12	30075959	30187436	25.08
62	a0007B20	30824142	30835981	8.00
63	a0036J15	31222238	31387974	13.86
64	a0037D16	31313973	31498115	24.11
65	a0018G20	32094917	32274028	12.54
66	b0024I19	32507293	32633286	0.00
67	b0014O02	33010624	33123772	0.00
68	a0024G16	33587038	33745554	0.00
69	b0037O03	33832455	34023561	20.87
70	a0004L01	34316249	34466638	15.53

Bd1 long arm				
**No.**	**Clone ID**	**Start [bp]**	**End [bp]**	**Repeat content [%]**
1	b0011C11	38625084	38768816	25.98
2	a0002G19	39219901	39352626	20.11
3	a0030K01	39352642	39424325	13.60
4	a0002I22	39952805	40102980	19.01
5	b0023K21	40363127	40508554	24.17
6	a0003G01	41070484	41199669	5.68
7	a0017I18	41400831	41536057	10.35
8	a0006H08	42291978	42445395	30.82
9	a0034B17	42516220	42665540	26.89
10	a0022M24	43211660	43355407	17.71
11	b0004P09	43536825	43670757	0.00
12	b0003O14	43968448	44100412	0.00
13	a0046B04	44701450	44835069	28.04
14	a0009H21	45130478	45275483	30.09
15	b0019N18	45624834	45763826	29.21
16	b0039A23	45904934	46085862	34.61
17	b0025P22	46564769	46692954	10.03
18	a0045K11	47017729	47159607	14.08
19	a0018I01	47327837	47472575	14.80
20	a0018A03	48153393	48351371	12.13
21	b0044L08	48612347	48783561	9.01
22	a0016L07	49131764	49282548	17.24
23	b0042L08	49567141	49756854	22.48
24	a0003G14	50139085	50274374	17.12
25	a0007A17	50488742	50629310	23.81
26	a0002G03	50987420	51131768	21.02
27	a0002M19	51404954	51599184	13.45
28	b0035K24	51720482	51914140	15.53
29	a0046C24	52577876	52717406	12.29
30	a0020C13	52998818	53110321	14.83
31	a0011F10	53395079	53532078	10.55
32	a0046G17	54082210	54253048	0.00
33	b0047M09	54775761	54934862	11.12
34	b0036M23	55099293	55274619	14.70
35	a0011D03	55361479	55401129	7.86
36	b0044C20	56154348	56285508	16.71
37	b0013C18	56402015	56523891	0.00
38	b0028P17	57093738	57225377	0.00
39	a0022N20	57208701	57348499	11.96
40	a0019B04	58011832	58155264	19.18

Bd1 long arm				
**No.**	**Clone ID**	**Start [bp]**	**End [bp]**	**Repeat content [%]**
41	b0003K21	58350464	58480351	15.82
42	a0010A14	59503419	59676696	0.00
43	a0008E12	60079495	60251020	17.16
44	a0005H16	60258365	60413281	0.00
45	b0037A14	61095306	61288697	19.17
46	a0012H18	61475648	61619144	0.00
47	b0003A21	61920716	62077069	0.00
48	a0034M17	62501248	62642447	0.00
49	a0045D19	63062019	63221983	14.31
50	b0022G04	63557791	63711230	17.60
51	a0013D23	64120769	64297730	16.02
52	b0011I02	64559074	64702171	27.84
53	a0003I14	65067565	65202176	23.09
54	a0046P14	65376014	65522455	21.81
55	a0009I15	65946210	66098108	21.98
56	b0026H13	66197674	66346594	22.18
57	b0030D22	67065313	67205367	25.08
58	a0019B19	67392232	67529032	8.00
59	b0003K24	67945518	68072820	13.86
60	a0011O07	68533765	68686250	24.11
61	a0043A05	68898017	69053532	12.54
62	b0004O01	69023274	69164463	23.47
63	b0039M08	69966292	70146601	29.34
64	a0040G14	70435911	70578835	17.94
65	b0017K19	71146553	71281318	0.00
66	a0021F18	71455475	71597258	0.00
67	a0041A08	72027767	72181888	6.97
68	b0002N07	72465040	72619352	4.45
69	a0005K09	72948475	73083942	4.87
70	b0039K17	73601518	73740071	5.12
71	a0033F06	74020535	74180685	4.77
72	b0035K23	74475472	74659792	12.79

Supplementary Table S2. Characteristics of BAC clones used for the chromosome painting of Bd2.

Bd2 short arm				
**No.**	**Clone ID**	**Start [bp]**	**End [bp]**	**Repeat content [%]**
1	a0038A01	1022	132144	4.73
2	a0026H23	501743	631176	5.15
3	b0039C09	1311448	1507438	6.21
4	a0027K15	1864643	2004976	4.27
5	b0035C01	2500100	2659222	4.28
6	b0002F19	2858161	3010741	5.64
7	a0028O04	3492740	3587755	10.20
8	b0048M15	3999943	4170302	18.58
9	a0002P22	4319740	4509765	7.60
10	a0045F24	6004397	6146555	12.87
11	a0047M10	7007047	7144308	8.94
12	a0019E04	8843922	9006117	13.80
13	a0012B07	9006125	9148678	6.31
14	a0005E09	10380990	10507985	10.31
15	b0048L18	12984227	13000547	8.85
16	a0044D02	14006553	14195269	11.10
17	a0017D02	15866689	16021967	17.90
18	a0031J04	16021976	16162135	12.06
19	a0047D12	17856422	17996794	27.25
20	a0021H13	18176419	18323680	28.06
21	a0026K14	19861012	20005795	15.73
22	a0043C22	20005803	20143867	25.91
23	b0006D07	20880418	21008785	17.52
24	b0015N23	21980992	22118800	12.71
25	b0038L02	22509927	22639901	23.44
26	b0011O19	25393226	25510444	14.12

Bd2 long arm				
No.	Clone ID	Start [bp]	End [bp]	Repeat content [%]
1	b0031J17	33543920	33664342	15.14
2	a0014K11	34309867	34503922	8.53
3	b0003D21	35507055	35522066	19.01
4	b0007E06	36376507	36505573	18.82
5	b0022I07	38509106	38646001	9.09
6	a0024L09	38997728	39192842	8.39
7	b0031K20	39779799	39931474	5.43
8	b0016E24	39997753	40003453	1.65
9	a0043N06	41508421	41714064	18.78
10	a0008H07	42500887	42664133	14.08
11	b0018H13	42943153	43001509	9.64
12	a0029H05	43505258	43648404	13.83
13	a0009N24	44005924	44173470	13.71
14	b0041G17	44876290	45007631	13.75
15	b0031I09	46500135	46639653	7.51
16	b0040K17	47000159	47021312	4.78
17	b0019P09	48369110	48504229	3.43
18	b0041J21	49505774	49706051	9.11
19	b0038L04	50005019	50143082	5.40
20	b0012J01	51003290	51006240	0.00
21	a0031O24	52001822	52162247	4.16
22	a0036P06	52875522	53003468	5.61
23	a0027O24	53007896	53168487	8.87
24	b0040O17	53370233	53504052	4.56
25	b0036G07	53816466	54010118	3.84
26	b0027N08	54420021	54540398	9.29
27	b0047O03	55698147	55846468	5.48
28	a0038M22	56336703	56502216	4.16
29	a0038G14	57002804	57148130	7.24

Supplementary Table S3. Characteristics of BAC clones used for the chromosome painting of Bd3.

Bd3 short arm				
**No.**	**Clone ID**	**Start [bp]**	**End [bp]**	**Repeat content [%]**
1	a0019P17	353866	501536	12.96
2	a0001O14	501589	646912	10.33
3	b0028O16	856255	1007650	12.86
4	a0005B05	1000513	1146984	5.57
5	a0011O22	1330674	1507458	7.88
6	a0024P19	1507465	1643914	14.28
7	b0026H03	1872780	2007217	6.12
8	b0040M13	2318183	2506944	9.33
9	b0022F20	2505550	2655293	9.03
10	a0015A18	4001904	4157452	8.62
11	a0007G18	4853075	4999533	15.21
12	b0041N16	5017958	5207531	12.89
13	a0001N07	6854023	7004002	7.62
14	a0029A17	7006740	7159041	6.88
15	b0038O09	8001902	8135334	16.54
16	b0016A22	11505050	11712720	21.97
17	b0010J18	13993335	14131952	16.02
18	a0014K09	14869360	15008860	15.03
19	b0041F02	15002696	15181874	5.37
20	a0022G01	16038657	16055486	5.49
21	a0017B02	19002671	19154844	11.10
22	b0009N11	19496003	19508052	2.37
23	a0026C10	19503371	19692680	16.69
24	a0019E11	19840599	19994588	21.87
25	b0014A01	20363699	20508591	19.13
26	a0033D16	22106200	22299788	16.81

Bd3 long arm				
**No.**	**Clone ID**	**Start [bp]**	**End [bp]**	**Repeat content [%]**
1	b0002I18	29506741	29629956	11.75
2	a0036L01	31849467	32007174	15.96
3	b0007K04	34625943	34771746	18.56
4	b0032D13	36350054	36506048	18.24
5	b0011M04	36854229	37002472	5.83
6	b0025H02	37377041	37501983	7.77
7	b0035D02	37506114	37762639	19.54
8	b0047N08	40007444	40153099	19.42
9	a0047F20	40911246	41000063	9.54
10	b0036D03	41006469	41221676	17.42
11	b0015F15	41505264	41681278	7.27
12	a0013E06	42292206	42500698	12.33
13	a0033K23	42848637	43004766	13.13
14	a0043C19	43854818	44001044	17.15
15	a0038N13	44001051	44142538	18.64
16	a0005G11	47463732	47500906	8.93
17	a0043A16	48007234	48184206	19.18
18	a0026K12	48876166	49007415	8.82
19	a0026M18	49347850	49503810	4.84
20	a0018C17	49869712	50007626	7.58
21	a0019B17	50354409	50508627	7.22
22	a0025I07	50508637	50670776	9.56
23	a0037F15	50854746	51004145	4.69
24	a0018O04	52001207	52171186	16.46
25	a0023N06	52300422	52503409	11.71
26	a0037C10	52501229	52687354	9.66
27	a0024K02	53356232	53501555	12.47
28	a0030K10	54003049	54158281	4.71
29	a0028B03	54337885	54500593	16.02
30	a0002F20	54500603	54637896	3.09
31	a0008G22	55503533	55665230	5.31
32	a0043F22	56008431	56171010	19.64
33	a0044B21	56862681	57005129	9.17
34	b0034A10	57009259	57107717	7.19
35	b0037C16	57302467	57322111	1.45
36	a0008L06	57499776	57533242	7.41
37	a0020N10	57504387	57653389	5.12

Supplementary Table S4. Characteristics of BAC clones used for the chromosome painting of Bd4.

Bd4 short arm				
**No.**	**Clone ID**	**Start [bp]**	**End [bp]**	**Repeat content [%]**
1	b0022F16	1375208	1513070	17.05
2	a0029D03	1509327	1527857	14.80
3	b0011J07	1877049	2006504	13.50
4	b0030B12	2007584	2157984	7.19
5	a0008M09	3005577	3057490	10.02
6	b0031P08	4003479	4005284	0.00
7	b0020L19	4509871	4652127	17.87
8	b0015K04	5000667	5192928	12.52
9	b0015K04	5192351	5334608	20.17
10	a0021K11	5356098	5506730	12.85
11	b0040J03	7830905	8001843	19.13
12	b0021B09	9502901	9667864	12.35
13	a0039N16	9858184	10002657	14.32
14	a0043D11	11006774	11150531	22.28
15	a0038M23	13356537	13506604	23.57
16	a0004L13	13506625	13718798	11.46
17	a0010I18	14002249	14164264	10.04
18	a0028M13	14467770	14501426	10.11
19	a0006A19	15500025	15633790	10.64
20	b0023J07	18378813	18509732	28.56

Bd4 long arm				
**No.**	**Clone ID**	**Start [bp]**	**End [bp]**	**Repeat content [%]**
1	a0047K04	27639991	27795427	18.44
2	b0012C05	28999425	29071930	12.74
3	a0006J17	29358544	29516826	9.42
4	b0027J13	30855184	31004161	28.40
5	a0013N14	31000455	31132079	9.47
6	a0020D08	32504625	32642850	8.94
7	b0033J04	32835278	33001029	24.02
8	a0029H13	33008088	33024284	20.44
9	b0005K02	33865813	34006488	20.04
10	b0002F10	34027870	34141505	10.19

Bd4 long arm				
**No.**	**Clone ID**	**Start [bp]**	**End [bp]**	**Repeat content [%]**
11	a0021F10	35000545	35176627	26.57
12	a0011F18	36857616	37009036	26.55
13	a0031C04	37506966	37653676	24.29
14	a0004J07	38767257	38918272	6.73
15	b0021H03	38925520	39066237	14.28
16	b0035E05	39350118	39526113	6.40
17	b0014B09	39506174	39642910	17.79
18	a0024O22	39853958	40006315	16.05
19	b0041J05	40005710	40200691	17.39
20	a0032J21	40499878	40546546	5.53
21	a0003H15	40835257	41003446	12.86
22	b0047A04	41006415	41014781	22.45
23	a0047P14	41364258	41502074	13.90
24	a0026J09	41502088	41633709	24.33
25	b0032E02	41852352	42005820	21.66
26	b0026E20	42346424	42505268	15.02
27	b0038H23	42789149	43003220	6.17
28	b0023G20	43352742	43500050	9.76
29	b0019I11	43876622	44010479	6.16
30	a0004A24	44343043	44510220	7.93
31	a0043N14	45504848	45661980	6.49
32	b0043F05	46001501	46132046	15.43
33	a0030C04	46357221	46502222	7.16
34	a0004O19	46502230	46658753	9.01
35	b0031I03	46846042	47008130	20.21
36	b0026N15	47003712	47134381	8.51
37	a0017H17	47506772	47643218	6.97
38	a0024E12	48004859	48154124	3.07
39	a0041I03	48350055	48507632	9.05

Supplementary Table S5. Characteristics of BAC clones used for the chromosome painting of Bd5.

Bd5 short arm				
**No.**	**Clone ID**	**Start [bp]**	**End [bp]**	**Repeat content [%]**
1	a0018K07	853205	1009526	16.20
2	a0019O20	1091367	1236179	16.00
3	b0041L07	2001626	2206122	23.87
4	b0010D07	2501377	2672539	21.99

Bd5 long arm				
**No.**	**Clone ID**	**Start [bp]**	**End [bp]**	**Repeat content [%]**
1	a0045F23	13499779	13653343	18.89
2	b0030K21	15304885	15505515	22.56
3	a0026M04	17499731	17633162	23.72
4	a0023L21	17634500	17679830	4.89
5	a0001F13	17802975	18003203	23.52
6	a0017D24	18003221	18155770	16.96
7	b0042J14	18312982	18503857	19.62
8	a0046O09	20358624	20503060	24.99
9	b0024J19	20845837	21003148	11.02
10	b0033K07	21003184	21110356	11.5
11	b0037B05	21507488	21710347	23.77
12	b0016H11	21877774	22006306	16.69
13	b0041K21	23480858	23500119	1.39
14	b0032J06	23870997	24003288	6.19
15	a0045J11	24003128	24154997	9.3
16	a0026B16	24841254	25002060	5.96
17	a0031B15	25503136	25695070	4.94
18	a0023B07	25746075	25906029	8.99
19	a0019J13	25906054	26098440	3.35
